# Cryptotanshinone attenuates LPS-induced acute lung injury by regulating metabolic reprogramming of macrophage

**DOI:** 10.3389/fmed.2022.1075465

**Published:** 2023-01-13

**Authors:** Zesen Ye, Panxia Wang, Guodong Feng, Quan Wang, Cui Liu, Jing Lu, Jianwen Chen, Peiqing Liu

**Affiliations:** ^1^Laboratory of Pharmacology and Toxicology, National-Local Joint Engineering Laboratory of Druggability and New Drugs Evaluation, Guangdong Province Engineering Laboratory for Druggability and New Drug Evaluation, School of Pharmaceutical Sciences, Sun Yat-sen University, Guangzhou, China; ^2^School of Pharmaceutical Science, Guangzhou Medical University, Guangzhou, China

**Keywords:** acute lung injury, Cryptotanshinone, macrophage polarization, metabolic reprogramming, AMPK

## Abstract

**Background:**

Acute lung injury (ALI) is a life-threatening inflammatory disease without effective therapeutic regimen. Macrophage polarization plays a key role in the initiation and resolution of pulmonary inflammation. Therefore, modulating macrophage phenotype is a potentially effective way for acute lung injury. Cryptotanshinone (CTS) is a lipophilic bioactive compound extracted from the root of *Salvia miltiorrhiza* with a variety of pharmacological effects, especially the anti-inflammatory role. In this study, we investigated the therapeutic and immunomodulatory effects of CTS on ALI.

**Materials and methods:**

The rat model of ALI was established by intratracheal instillation of LPS (5 mg/kg) to evaluate the lung protective effect of CTS *in vivo* and to explore the regulation of CTS on the phenotype of lung macrophage polarization. LPS (1 μg/mL) was used to stimulate RAW264.7 macrophages *in vitro* to further explore the effect of CTS on the polarization and metabolic reprogramming of RAW264.7 macrophages and to clarify the potential mechanism of CTS anti-ALI.

**Results:**

CTS significantly improved lung function, reduced pulmonary edema, effectively inhibited pulmonary inflammatory infiltration, and alleviated ALI. Both *in vivo* and *in vitro* results revealed that CTS inhibited the differentiation of macrophage into the M1 phenotype and promoted polarization into M2 phenotype during ALI. Further *in vitro* studies indicated that CTS significantly suppressed LPS-induced metabolic transition from aerobic oxidation to glycolysis in macrophages. Mechanistically, CTS blocked LPS-induced metabolic transformation of macrophages by activating AMPK.

**Conclusion:**

These findings demonstrated that CTS regulates macrophage metabolism by activating AMPK, and then induced M1-type macrophages to transform into M2-type macrophages, thereby alleviating the inflammatory response of ALI, suggesting that CTS might be a potential anti-ALI agent.

## 1. Introduction

Acute lung injury (ALI) is a life-threatening respiratory disease which can lead to respiratory failure and higher mortality (1). The main pathogenesis of ALI are sharp increase in pulmonary inflammatory responses, diffused alveolar injury and pulmonary edema, which might ultimately lead to acute hypoxemia (2). Currently, the clinical treatment for ALI is limited and specific drugs for ALI are also still lacking (3). Even though mechanical ventilation could partially relieve the pathology of ALI, long-term mechanical ventilation always increases ventilator-related lung injury, higher mortality and heavy financial burden (4, 5). Therefore, it is urgent to explore new strategies to improve ALI.

As the core participants of innate immune response, alveolar macrophages play a pivotal role in the initiation, development and resolution of lung inflammation during acute lung injury (6). In the early exudative stage of ALI, stimulated by Th1-type cytokines such as TNF-α or interferon, macrophages could differentiated into M1 phenotype or proinflammatory macrophages to mediate inflammatory responses via releasing proinflammatory cytokines and chemokines (7). During the repair phase of ALI, activated by Th2-type cytokines, such as IL-4, IL-13 and immune complex, macrophages prefer to polarize into M2 phenotype or anti-inflammatory macrophages (6). Macrophages are key orchestrators in the progress of ALI and modulating the phenotype of macrophage might improve the prognosis of ALI.

Furthermore, accumulating evidence suggests that metabolic reprogramming plays a crucial role in the differentiation of macrophages (8). As indicated by the increasing glucose uptake and lactic acid production, activated M1 phenotypes are highly dependent on aerobic glycolysis to meet energy requirements for rapid proliferation and cytokine production (9). Conversely, M2 phenotypes are mainly dependent on mitochondrial oxidative phosphorylation and fatty acid oxidation for energy supplement (10). The metabolic reprogramming is not only to meet the energy requirements of macrophages in response to vary stimulus, but also a necessary step to drive macrophage polarization (8). Previous studies have shown that overexpression of glucose transporter 1 (GLUT1), a key gene involved in glycolysis, could drive macrophages differentiated into M1 phenotype by promoting glycolysis (11). However, 2-DG (2-deoxy-D-glucose), a well-established inhibitor of glycolysis, inhibits the proinflammatory phenotype of M1 macrophages by blocking glycolysis (12). Additionally, knockout of genes related to fatty acid metabolism or mitochondrial oxidative phosphorylation blocked the activation of M2 phenotype (13, 14). Therefore, intervention of the metabolic pattern of macrophages will control the phenotype of macrophages and might play a pivotal role in ALI.

Cryptotanshinone (CTS) is extracted from *Salvia miltiorrhiza* and belongs to diterpenoid quinones with a variety of pharmacological activities such as anti-inflammatory, anti-cancer, anti-oxidant and anti-fibrosis (15). Previous studies from other’s and our laboratory have systematically studied the effects of CTS on arthritis (16), atherosclerosis (17), and Alzheimer’s disease (18), all of which indicated the excellent therapeutic effects of CTS. Previously, we have reported that CTS effectively protected lung from pulmonary fibrosis by inhibiting Smad and STAT3 signaling pathways (19). CTS inhibited the occurrence and development of acute colitis and cerebral ischemic stroke by promoting the trans-differentiation of M1 phenotype into the M2 phenotype (20, 21). In addition, CTS has been shown to exert anticancer effects by blocking glycolysis to inhibit tumor cell proliferation and migration (22, 23). Even though CTS could inhibited the progression of protect ALI by inhibiting NF-κB signal pathway (24), it was still unknown whether CTS could alleviate the inflammatory response ALI by altering the metabolic pattern of macrophages.

In this study, we found that CTS effectively improved pulmonary function and relieved LPS-induced pulmonary inflammation in rats with ALI. This study further revealed that CTS inhibited the accumulation of the M1 phenotype (pro-inflammatory) macrophage and increased the M2 phenotype (anti-inflammatory) macrophage in the lung tissue. Additionally, both the *in vivo* and *in vitro* results showed that CTS could regulate metabolic reprogramming of macrophage by activating AMPK.

## 2. Materials and methods

### 2.1. Reagents

CTS (purity ≥ 98%) were obtained from the Laboratory of Pharmacology and Toxicology, School of Pharmaceutical Sciences, Sun Yat-sen University (Guangzhou, China). LPS was purchased from Sigma-Aldrich (St. Louis, USA). Compound C were purchased from Selleck (Shanghai, China). Dulbecco’s modified Eagle’s medium (DMEM) was purchased from Gibco (NY, USA). Fetal bovine serum (FBS) was abtained from HyClone (Logan, USA). Myeloperoxidase (MPO) and lactic acid assay kits were from Nanjing Jiancheng Bioengineering Institute (Nanjing, China). The antibodies against CD86 (A1199) and Arg-1 (A4923) were obtained from ABclonal (Wuhan, China). The antibodies against CD206 (ab64693) were purchased from Abcam (Cambridge, MA). The antibody against iNOS (AF0199) was from Affinity Biosciences (OH, United States). The antibodies against β-actin (6600-1-Ig), GLUT1 66290-1-Ig) and PFKFB3 (3763-1-AP) were purchased from Proteintech (Chicago, USA). The antibodies against PKM2 (D78A4), HIF-1α (D1S7W), p-AMPK (40H9) and AMPK (2532) were obtained from Cell Signaling Technology (Danvers, USA). The antibodies for flow cytometry, including phycoerythrin (PE) anti- CD86 (105007) and allophycocyanin (APC) anti-CD206 (141708) were obtained from BioLegend (San Diego, USA).

### 2.2. LPS - Induced acute lung injury rat model

The animal procedures were approved by the Research Ethics Committee of Sun Yat-sen University and conducted following the Guide for the Care and Use of Laboratory (NIH Publication No. 85-23, revised 1996). Sprague-Dawley rats (SD rats, male, SPF grade, 6-8 weeks, weighing 200-230 g) were supplied by the Experimental Animal Center of Sun Yat-sen University (Guangzhou, China) and the certification No. 44008500024762. LPS (5 mg/kg) was dissolved in normal saline (NS) and administrated by intratracheal instillation to SD and the acute lung injury model was established by LPS administration for 24 h. SD rats were randomly divided into several groups (10 rats in each group): the control group, the LPS-induced acute lung injury model group and the CTS treatment groups at three different concentrations. CTS was dissolved in sodium carboxymethylcellulose (CMC-Na, 5%, W/V) at different concentrations (15, 30 and 60 mg/kg/day). Before LPS treatment, the CTS treatment group was pre-administered intragastrically for 5 days, while the control and model groups were given the same volume of solvent solution. The rats in each drug administration group were given drug intervention once at 6 hours, 12 hours and 18 hours after modeling.

### 2.3. Pulmonary function assessment

Pulmonary function was measured by using a whole-body plethysmograph (Emka Technologies, Paris, France) for rats. The parameters of pulmonary function included enhanced pause (Penh), relaxation time (RT), end inspiratory pause (EIP), end expiratory pause (EEP) and minute ventilation volume (MV). Rats were placed in a plethysmograph chamber and 10 min was used for acclimation before 5 min of assessing respiratory parameters.

### 2.4. Histopathological assessment and the measurement of lung wet/dry (W/D) weight ratio

At the end of the *in vivo* experiment, all rats were anesthetized and sacrificed. The whole lung of the rat was quickly removed and weighed. The lung weight to body weight ratios were calculated according to the following formula: Lung to Body weight ratio = (Lung weight (g))/(Body weight (g)) × 100%. Subsequently, left lung tissue was fixed in 4% paraformaldehyde, embedded in paraffin, sectionalized, and stained with hematoxylin and eosin (HE). HE scores were calculated by light microscopic analysis of four parameters including alveolar septal thickness, interstitial edema, infiltration of inflammatory cells, and alveolar congestion/collapse. Each parameter was categorized into four grades: 0 = normal; 1 ≤ 25%; 2 = 25–50%; 3 = 50–75%; and 4 ≥ 75%, and the mean score of the four parameters was used to represent the overall lung injury (25). Histopathological images were captured and analysis by using light microscope at 400x magnification (EVOS FL Auto Cell Imaging System, USA). The right lung was excised and weighed to assay wet weight, followed by drying at 80 ? for 48 h to obtain dry weight. The lung wet/dry (W/D) weight ratio W/D weight ratio was calculated to indicate pulmonary edema formation.

### 2.5. Measurement of myeloperoxidase (MPO) activity

MPO activity of lung tissue was measured by a commercial kit according to the manufacturer’s instructions (A044, Nanjing Jiancheng Bioengineering Institute, China).

### 2.6. Bronchoalveolar lavage fluid (BALF) collection

By intratracheal injection of 5 mL sterile saline and then slowly withdrawn, repeated irrigation three times. The collected bronchoalveolar lavage fluid was centrifuged at 300 g for 10 min at 4°C, and the supernatant was extracted for subsequent cytokine and total protein inspection. Cytokine levels in the supernatants of BALF were determined using commercially available TNF-α, IL-1β, IL-6, IL-10 ELISA kits (Wuhan Huamei Biotech Co., Ltd., Wuhan, China) according to the manufacturer’s instructions. Total protein concentration in the supernatant BALF was determined using the BCA protein quantification kit (Thermo Fisher Scientific, Waltham, USA).

### 2.7. Cell culture

RAW264.7 cell line was obtained from ProCell (Wuhan, China) and maintained in 37°C incubators with 5% CO_2_. The cultured media was DMEM media with 10% fetal bovine serum, 100 U/ml penicillin and 100 mg/ml streptomycin. RAW264.7 were pretreated with the indicated concentrations of CTS (2.5, 5, 10 μM) for 2 h before being stimulated with LPS (1 μg/mL) for another 24 h.

### 2.8. Measurement of glucose uptake and lactic acid in RAW264.7

Glucose uptake of RAW264.7 cells were assayed by using the Glucose Uptake-Glo Assay kit from Promega (Wisconsin, USA). The level of lacticte acid was detected by using a commercial kit (Nanjing Jiancheng Bioengineering Institute, China).

### 2.9. Immunofluorescence staining

Frozen lung tissue sections were fixed in acetone for 20 min, then permeabilized by 0.3% Triton X-100 (Sigma, St. Louis, USA) for 15 min and blocked by 10% goat serum for 30 min. Subsequently, sections were incubated with anti-CD68 antibody (BIO-RAD, MCA341GA 1:100 dilution) overnight at 4°C, anti-CD86 antibody (ABclonal, A11991, 100 dilution), and anti-CD206 antibody respectively (Abcam, ab64693, 1:100 dilution). Sections were washed with PBS followed by incubation with fluorescent secondary antibodies (Abcam, ab150116, ab150077) in dark for 1 h at room temperature. Finally, the sections were re-stained with 4’,6-diamidino-2-phenylindole (DAPI) for 10 min at room temperature. Fluorescent images were captured under a fluorescent microscope (EVOS FL Auto Cell Imaging System, USA).

### 2.10. Immunohistochemical staining

For immunohistochemistry (IHC) analysis, paraffin-embedded lung tissues were deparaffinized, rehydrated through an alcohol series followed by antigen retrieval with sodium citrate buffer. Tumor sections were blocked with 5% normal goat serum with 0.3% Triton X-100 and 3% H_2_O_2_ in PBS for 60 min at room temperature and then incubated with anti-iNOS antibody (Affinity Biosciences, AF0199, 1:100 dilution) or anti-Arg-1 antibody (ABclonal, A4923, 1:100 dilution) at 4°C overnight. Then, HRP-conjugated goat anti-rabbit IgG polyclonal antibody (Abcam, ab6721, 1:1000) were used. Alternatively, sections were stained with DAB and restained with hematoxylin, and then photographed using a microscope (EVOS FL Auto Cell Imaging System, USA). The area of the positive area was calculated using Image-Pro Plus 6.0 software (Media Cyber??netics, Silver Spring, USA).

### 2.11. CCK-8 assay

RAW264.7 cells were grown in 96-well plates at a density of 10000 cells per well and cultured overnight in the incubator. Different concentrations of CTS (2.5, 5, 10 μM) were administrated to cells for 24 h with or without LPS (1 μg/mL) stimulation. Subsequently, 10 μL of CCK-8 solution was added into each well and incubated for another 4 h. The absorbance values of each well were measured at 450 nm using a microplate reader (Bio-Tek, Winooski, USA).

### 2.12. Flow cytometry analysis

RAW264.7 cell suspension was collected and incubated with anti-CD16/32 (BioLegend, San Diego, USA) at 4 ? for 20 min to block Fc receptor. And then, the cells were washed twice in staining buffer (BioLegend, San Diego, USA) and stained with anti-CD86-PE antibody (BioLegend, San Diego, USA)) or anti-CD206-APC antibody (BioLegend, San Diego, USA) for 30 min. Followed by washing twice with staining buffer (BioLegend, San Diego, USA), resuspended the RAW264.7 cells in 300 μL staining buffer. Flow cytometry data were obtained using a CytoFLEX S flow cytometer (Beckman Coutler, Brea, USA) and analyzed using FlowJo software (Ashland, USA).

### 2.13. Measurement of oxygen consumption rate (OCR) and extracellular acidification rate (ECAR) of RAW264.7 cells

OCR and ECAR were measured by using XF-96 Extracellular flux analyzer (Seahorse Bioscience, North Billerica, MA, USA) to assess mitochondrial oxidative phosphorylation and glycolysis capacity respectively. CTS at different concentrations (2.5, 5, 10 μM) were pre-incubated with RAW264.7 cells for 2 h with co-stimulation of LPS (1 μg/mL) for 24 h. For the ECAR assay, the medium was replaced with XF solution containing 2 mmol/L L-glutamine prior to analysis. Glucose (10 mM), oligomycin (1 μM) and 2-DG (50 mM) were used to determine the glycolysis rate, glycolysis capacity and glycolysis reserve capacity of cells. For the OCR assay, the medium was replaced with XF solution containing glucose (2.5 M), pyruvate (1 mM) and glutamine (1 mM) prior to analysis. Oligomycin (1 μM), FCCP (0.75 μM), rotenone (0.5 μM) and antimycin A (0.5 μM) were used to determine basal respiration, mitochondrial ATP production and maximum respiration.

### 2.14. Protein extraction and western blot

Total protein was extracted from lung tissues or RAW264.7 cells using RIPA lysis buffers containing protease inhibitors and phosphatase inhibitors. The concentrations of proteins were measured using the BCA protein quantification kit (Thermo Fisher Scientific, Waltham, USA). Equal amount of protein samples was boiled and loaded in 10% SDS-PAGE for separation and transferred to PVDF membrane (Meck Millipore, Burlington, USA). The PVDF membranes were blocked with 5% skim milk at room temperature for 1 hour and then incubated with different primary antibodies at 4°C overnight. The membranes were incubated with the corresponding secondary antibodies for 1 h at room temperature. The proteins were visualized by chemiluminescence using an ECL system (GE Healthcare, Pittsburgh, USA) and the images were captured using an imaging system (Tanon, Shanghai, China).

### 2.15. Real-time polymerase chain reaction (RT-PCR)

Total RNA was extracted from RAW264.7 cells using Trizol reagent (Invitrogen, Carlsbad, USA) and cDNA was synthesized using the QuantiTect reverse transcription kit (QIAGEN, Valencia, USA) according to the manufacturer’s protocol. The relative mRNA expression level was determined using the 2-delta delta Ct analysis method, where GAPDH was used as a home keeper gene. The primer sequences used in this experiment were listed in Table 1.

**TABLE 1:** RT-qPCR Primers used in this study.

Primer		Sequence (5′–3′)
GLUT1	Forward Reverse	ACGATCTGAGCTACGGGGT GCCCGTCACCTTCTTGCTG
PFKFB3	Forward Reverse	CAACTCCCCAACCGTGATTGT TGAGGTAGCGAGTCAGCTTCT
PKM2	Forward Reverse	ATTACCAGCGACCCCACAGAA ACGGCATCCTTACACAGCACA
CPT1A	Forward Reverse	TATGGTCAAGGTCTTCTCGGGTCG AGTGCTGTCATGCGTTGGAAGTCTC
CPT2	Forward Reverse	TCGGCCCTTAAGTGCTGTCT TTTAGGGATAGGCAGCCTGGG
MCAD	Forward Reverse	TGACAAAAGCGGGGAGTACC GCACCCCTGTACACCCATAC
GAPDH	Forward Reverse	ACCCTTAAGAGGGATGCTGC CCCAATACGGCCAAATCCGT

### 2.16. Statistical analysis

The results were expressed as mean ± standard error of mean (SEM) from at least three independent experiments and analyzed by using GraphPad Prism 8.0 software (San Diego, CA, USA). Student’s t-test was used to compare differences between two groups. Differences between groups were compared using one-way analysis of variance (ANOVA) followed by *Post hoc* Bonferroni’s test. *P* < 0.05 was considered statistically significant.

## 3. Results

### 3.1. CTS ameliorated LPS-induced acute lung injury in rats

**FIGURE 1:**
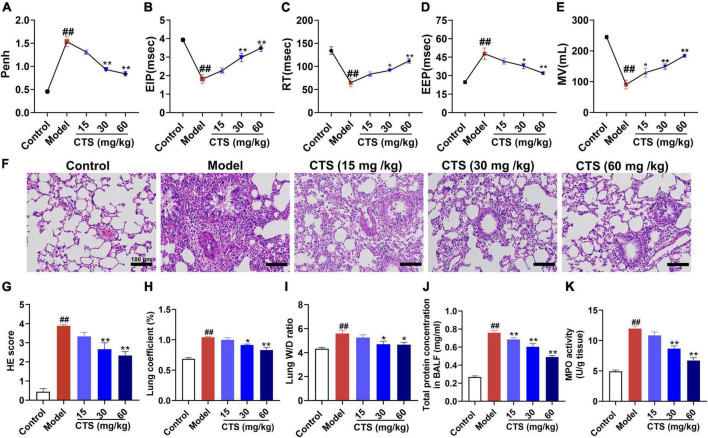
Cryptotanshinone (CTS) ameliorates LPS-induced acute lung injury in rats. LPS was used to induce *in vivo* ALI and CTS at different concentrations was administrated to rat. Representative parameters of mice pulmonary function: **(A)** enhanced pause (Penh), **(B)** end-inspiratory pause (EIP), **(C)** relaxation time (RT), **(D)** end-expiratory pause (EEP), **(E)** minute ventilation volume (MV), *n* = 6. **(F)** Representative HE staining results of lung histopathological changes, scale bar: 100 μm, *n* = 6. **(G)** Lung histopathological score, *n* = 6. **(H)** Lung coefficient **(%)** changes in each group, *n* = 8. **(I)** Wet-dry weight ratio of right lung (Lung W/D ratio) in rats, *n* = 6. **(J)** Total protein concentration in BALF, *n* = 5. **(K)** The MPO activity level in the lung tissues was measured, *n* = 6. ^##^*P* < 0.01 vs. the control group; *P < 0.05, **P < 0.01 vs. the Model group.

To determine the effects of CTS on pulmonary function of rats, non-invasive lung function tests were performed in rats. Penh is an indicator of airway resistance to positively reflect the constriction degree of internal bronchi. Compared with control group, a single dose of LPS (5 mg/kg) administration through intratracheal instillation significantly increased the value of enhanced pause (Penh) (Figure 1A), shortened the time period of end-inspiratory pause time (EIP) and relaxation time (RT) (Figures 1B, C), prolonged end-expiratory pause time (EEP) (Figure 1D), and finally decreased minute ventilation volume (MV) (Figure 1E). These results indicated that LPS administration successfully induced acute lung injury of rats. In contrast, CTS treatment at 15, 30 and 60 mg/kg effectively improved pulmonary function of rat in a dose-dependent manner (Figures 1A–E).

Additionally, pathological changes of lung tissue were analyzed by HE staining. As Figures 1F, G shown, the pulmonary structure was destroyed following LPS-stimulation as evidenced by disorganized pulmonary alveoli structure, obvious perivascular edema, widened space and significantly thickened alveolar walls accompanied by a large number of inflammatory cell infiltration, all of which were effectively relieved by CTS. Furthermore, LPS-induced increase in lung coefficient and right lung wet to dry weight ratio (Lung W/D ratio) were also relieved by CTS treatment in a dose dependent manner (Figures 1H, I). Total protein concentration in BALF and neutrophil infiltration was used to assess permeability of lung and the severity of pulmonary edema. Compared with the control group, LPS significantly induced the total protein concentration of BALF (Figure 1J). while CTS decreased the level of BALF in dose-dependent manner. In addition, LPS induced the neutrophil infiltration as indicated by the increased activity of MPO in lung tissue (Figure 1K). Moreover, CTS treatment relieved the protein concentration of BALF and neutrophil infiltration. All these results revealed that CTS alleviated lung pathology and inflammatory cell infiltration in a dose-dependent manner.

### 3.2. CTS inhibited inflammatory response by regulating macrophage polarization

**FIGURE 2:**
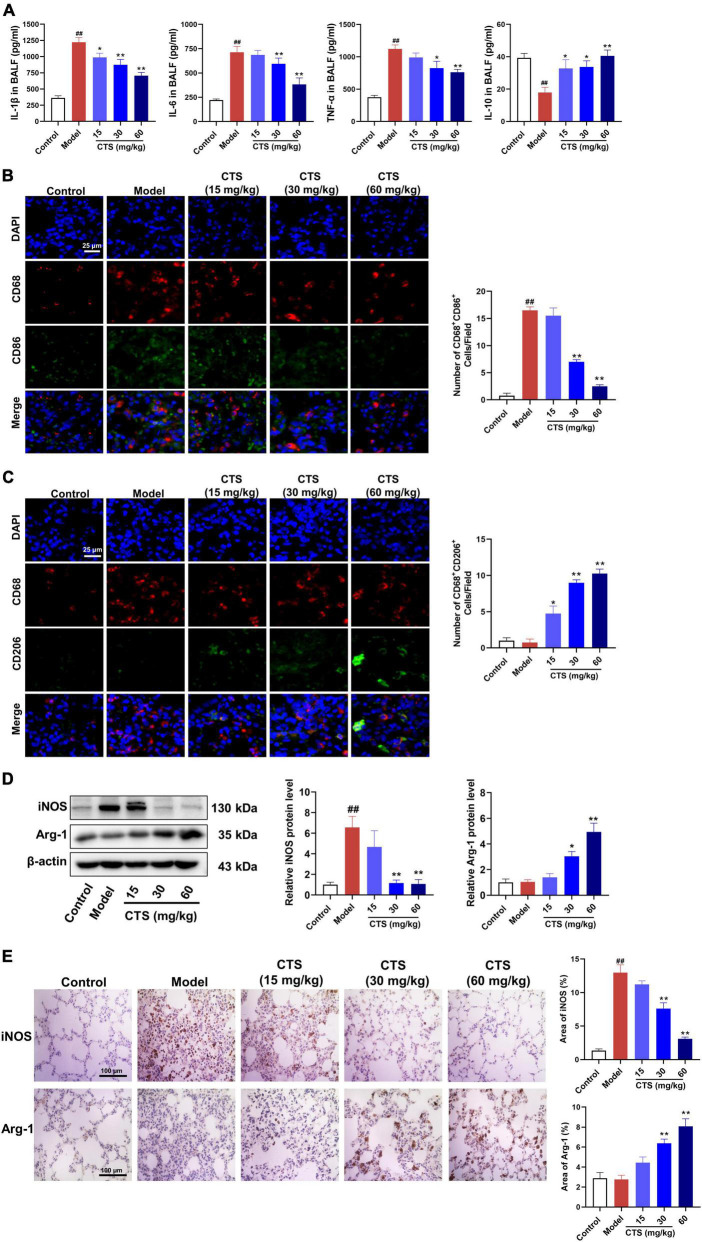
Cryptotanshinone (CTS) inhibited M1-type polarization and induces M2-type polarization of macrophages *in vivo*. **(A)** The levels of IL-1β, IL-6, TNF-α, and IL-10 in BALF were determined using ELISA, *n* = 6. **(B,C)** Representative immunofluorescence image of lung tissues. DAPI (blue), CD68 (red), CD86 (green), and CD206 (green), scales: 25 μm, *n* = 4. **(D)** The protein levels of iNOS and Arg-1, *n* = 4. **(E)** Lung sections were immunohistochemically stained by anti-iNOS antibody and anti-Arg-1 antibody, *n* = 4. Data were presented as the mean ± SEM. ^##^*P* < 0.01 vs. the control group; *P < 0.05, **P < 0.01 vs. the Model group.

Macrophages are sentinel cells of the lung innate immune system and can be differentiated into the proinflammatory (M1) phenotype or anti-inflammatory (M2) phenotype macrophages according to different stimulations (26, 27). Excessive activation of M1 phenotype macrophages or deficiency of M2 phenotype macrophages is the key factors causing uncontrolled lung inflammation in acute lung injury (28). To explore the *in vivo* effects of CTS on inflammatory response and macrophage polarization, we measured the levels of different cytokines in alveolar lavage fluid and the changes of macrophage polarization subtypes in lung tissue. Our ELISA results showed that LPS significantly induced the secretion of pro-inflammatory cytokines (such as IL-1β, IL-6 and TNF-α) and inhibited the secretion of anti-inflammatory factors (IL-10) in alveolar lavage fluid (Figure 2A). However, CTS significantly inhibited the secretion of IL-1β, IL-6 and TNF-α, and increased the IL-10 levels in alveolar lavage fluid (Figure 2A). Cluster of differentiation 68 (CD68) was used as a pan-macrophage marker, cluster of differentiation 86 (CD86) was used as a specific marker for M1 macrophages, and cluster of differentiation 206 (CD206) was used as a specific marker for M2 macrophages (29). Our immunofluorescence results showed that the number of CD68^+^CD86^+^ macrophages significantly increased during LPS-induced acute lung injury rats (Figures 2B, C). Western blotting and immunohistochemical also showed that the expression of iNOS was also increased following LPS-stimulation (Figures 2D, E). All these results indicated that LPS promoted macrophage differentiated into the proinflammatory type. However, we found that CTS could significantly promote the trans-differentiation of M1 macrophages into M2 macrophages, evidence by decreased CD68^+^CD86^+^ and iNOS level and increased the protein levels of CD68^+^CD206^+^ and Arg1 level.

### 3.3. CTS inhibited macrophage polarized to M1 type and induced to M2 type in RAW264.7 cell line

**FIGURE 3:**
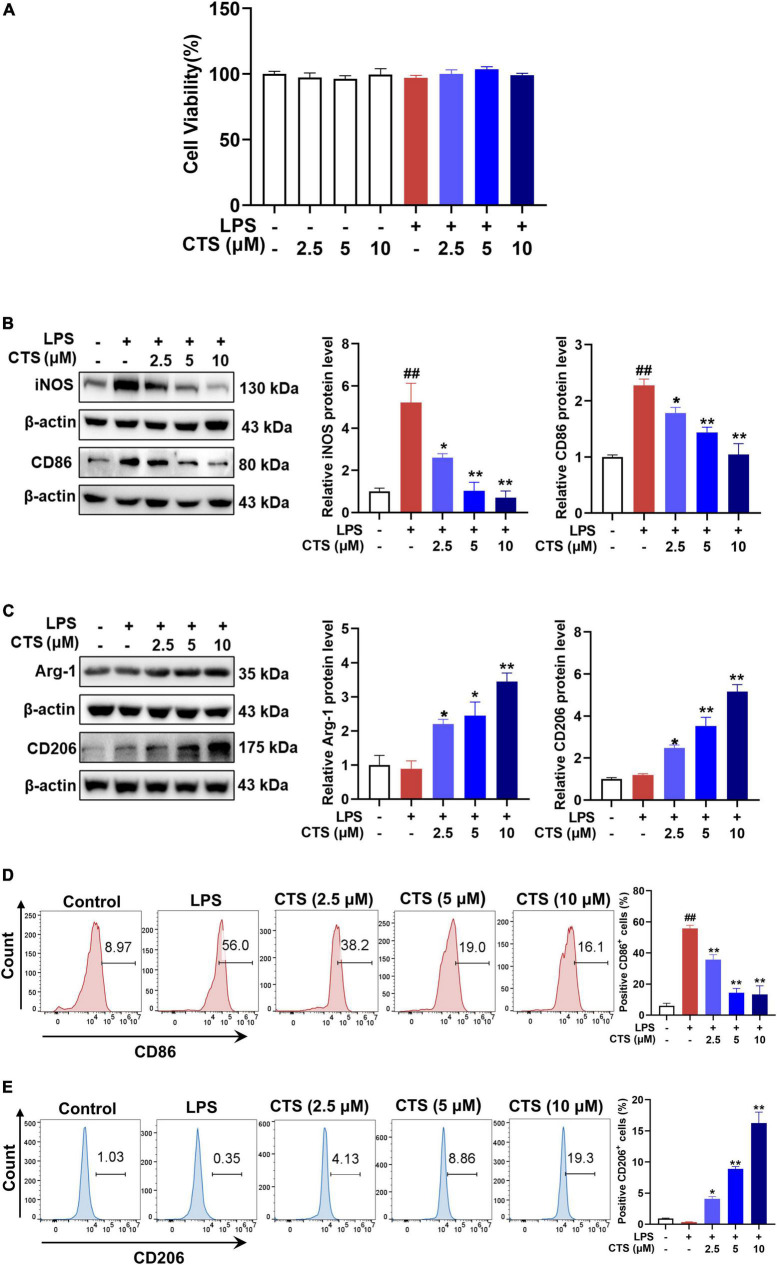
Cryptotanshinone (CTS) inhibited M1-type polarization and promoted M2-type polarization of RAW264.7 macrophages. **(A)** CTS alone or co-treatment with or without LPS stimulation and cell viability was measured. RAW264.7 were pretreated with CTS (2.5, 5, and 10 μM) for 2 h and then co-stimulated with LPS (1 μg/mL) for another 24 h. **(B)** The protein expression of iNOS and CD86, *n* = 3. **(C)** The protein expression of Arg-1 and CD206, *n* = 3. **(D)** The proportion of CD86 positive cells was analyzed by flow cytometry, *n* = 3. **(E)** The proportion of CD206 positive cells was analyzed by flow cytometry, *n* = 3. Data were presented as the mean ± SEM. ^##^*P* < 0.01 vs. the control group; *P < 0.05, **P < 0.01 vs. the LPS group.

Our *in vivo* results suggested that CTS inhibited LPS-induced inflammatory response of lung tissues by regulating macrophage polarization. Therefore, we further validated the effects of CTS on macrophage polarization by using RAW264.7 cell line. Firstly, CCK-8 results showed that CTS treatment with or without LPS did not alter the cell viability of RAW264.7 cell line (Figure 3A). According to our previous studies (30), CTS was used at different concentrations (2.5, 5, 10 μM) to inhibit inflammation. CTS could dose-dependently relieved LPS-induced expression of iNOS and CD86 (Figure 3B), and increased expression of Arg-1 and CD206 (Figure 3C). Our flow cytometry results furthermore showed that CTS dose-dependently decreased the proportion of CD86^+^ M1 macrophages and increased the proportion of CD206^+^ M2 macrophages (Figures 3D, E). Collectively, both *in vitro* and *in vivo* results consistently showed that CTS inhibited the polarization of macrophage and relieved inflammation *in vitro*.

### 3.4. CTS ameliorated LPS-induced metabolism dysfunction of macrophages

**FIGURE 4:**
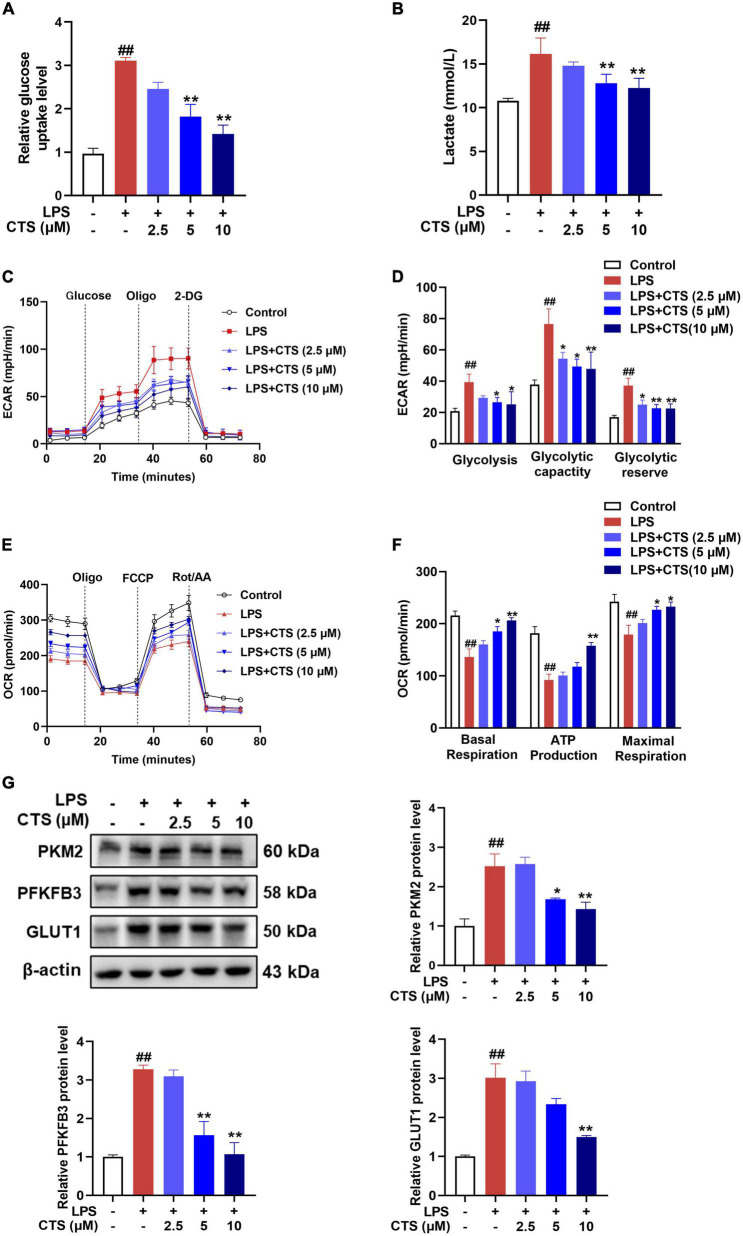
Cryptotanshinone (CTS) blocks LPS-induced metabolic reprogramming of RAW264.7 macrophages. RAW264.7 were pretreated with CTS (2.5, 5, and 10 μM) for 2 h and then co-treated with LPS for another 24 h. **(A)** Glucose uptake was detected using assay kit, *n* = 4. **(B)** The level of lactic acid, *n* = 4. **(C,D)** The extracellular acidification rate (ECAR) of macrophages were measured, *n* = 4. **(E,F)** The oxygen consumption rate (OCR) was measured, *n* = 4. **(G)** The protein levels of PKM2, PFKFB3 and GLUT1 were analyzed by using western blotting assay, *n* = 3. Data were presented as mean ± SEM. ^##^*P* < 0.01 vs. the control group; *P < 0.05, **P < 0.01 vs. the LPS group.

Phenotypic transformation of macrophages are closely related to the metabolism pattern (31). Based on metabolic characteristics of different macrophages phenotypes, M1 phenotype macrophages mainly rely on glycolysis, while M2 phenotype macrophages rely on fatty acid oxidation (FAO) and oxidative phosphorylation (OXPHOS) (32). Therefore, we further detected the effects of CTS on macrophage metabolism. Our results showed that CTS abrogated LPS-induced glucose uptake and lactic acid production in macrophage in a dose-dependent manner (Figures 4A, B). Extracellular acidification rate (ECAR) is a key indicator for measuring glycolysis flux and mitochondrial oxygen consumption rate (OCR) is the gold standard for detecting oxidative phosphorylation. Subsequently, we detected ECAR and OCR respectively in macrophage by using XF-96 extracellular flux analyzer. As shown in Figures 4C, D, LPS significantly increased glycolysis rate, glycolytic capacity and higher glycolysis reserve capacity, whereas basal respiration, mitochondrial related ATP production and maximum respiration rate were significantly inhibited in macrophage following LPS stimulation (Figures 4E, F). Conversely, CTS effectively relieved glycolysis and improved mitochondrial oxidative phosphorylation of macrophage (Figures 4C–F). Moreover, we detected the expression of proteins closely related to glycolysis such as pyruvate kinase M2 (PKM2), and 6-phosphofructo-2kinase/fructose-2,6-biphosphatase 3 (PFKFB3) and glucose transporter 1 (GLUT1). As shown in Figure 4G, the expression of PKM2, PFKFB3 and GLUT1 were significantly increased by LPS, whereas CTS treatment effectively inhibited the expression of these proteins. These results indicated that CTS might block LPS-induced metabolic dysfunction in macrophage.

### 3.5. AMPK was involved in the regulation of CTS on RAW264.7 macrophage polarization

**FIGURE 5:**
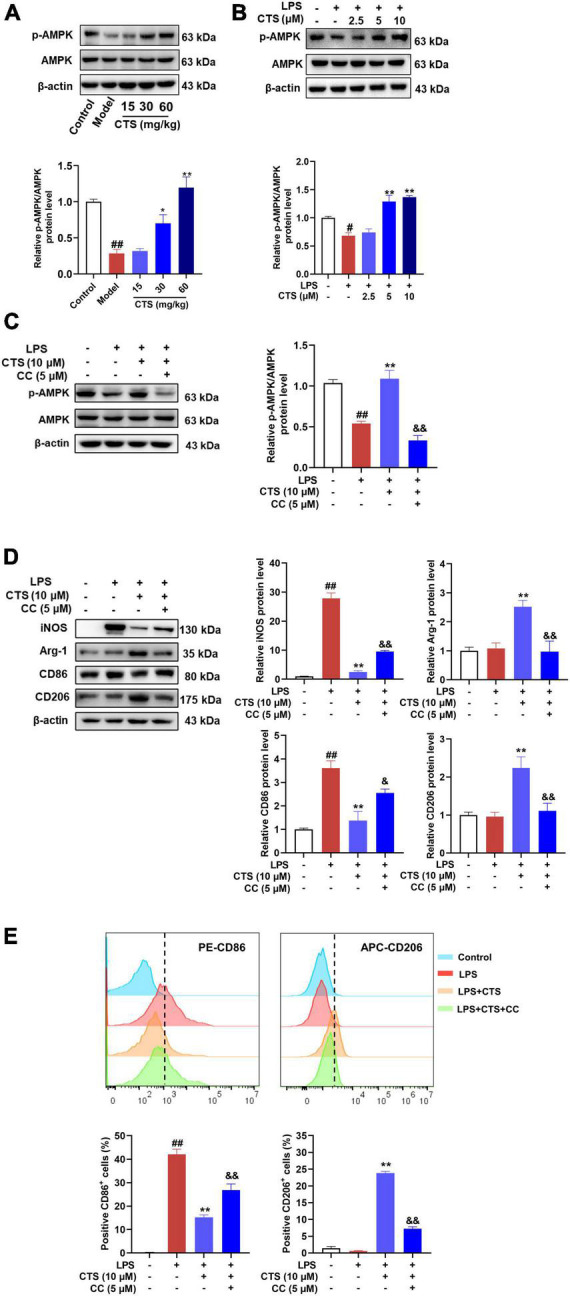
Cryptotanshinone (CTS) regulates RAW264.7 macrophage polarization via AMPK. **(A)** The protein levels of p-AMPK/AMPK in LPS-induced acute lung injury were analyzed by western blot, *n* = 4. **(B)** The protein expression of p-AMPK/AMPK in LPS-treated macrophage was analyzed by western blot, *n* = 3. **(C-E)** RAW264.7 pretreated with 5?μM AMPK inhibitor compound C for 2 h were treated with 10 μM CTS for 2?h and then exposed to LPS for another 24 h. **(C)** The protein expression levels of p-AMPK/AMPK were assessed by western blot analysis, *n* = 3. **(D)** The protein expression of iNOS, Arg-1, CD86, and CD206 was detected by Western blot, *n* = 3. **(E)** The proportion of CD86^+^ cells and CD206^+^ cells were analyzed by flow cytometry, *n* = 3. Data were presented as the mean ± SEM. ^#^*P* < 0.05, ^##^*P* < 0.01 vs. the control group; *P < 0.05, **P < 0.01 vs. the LPS group; ^&^*P* < 0.05, ^&&^*P* < 0.01 vs. the LPS + CTS (10 μM) group.

As a sensor of intracellular energy metabolism, AMP-activated protein kinase (AMPK) plays an important role in oxidative phosphorylation, cell growth and regulation of immune responses (33). AMPK has been shown to be a metabolic regulator of macrophage polarization (34), which was inhibited in LPS-induced M1 type macrophage and meant increased glycolysis as major metabolism pathway (35). However, activation of AMPK could switch the metabolism pattern from glycolysis to aerobic oxidation and promote the transformation of macrophages from M1 to M2 phenotype (36, 37). According to previous studies (38), CTS is an activator of AMPK pathway. Whether it could relieve LPS induced inflammation and metabolism dysfunction by activating AMPK remains unknown. Therefore, we detected the phosphorylation at ser172 and total protein level of AMPK in macrophage with CTS and LPS co-treatment. As shown in Figures 5A, B, both the *in vivo* and *in vitro* results showed that the phosphorylation of AMPK was decreased following LPS stimulation, whereas CTS treatment significantly augmented AMPK ser127 phosphorylation (Figures 5A, B). These results indicated that CTS activated AMPK during ALI.

Compound C, a specific pharmacological inhibitor for AMPK, was used to further verify the involvement of AMPK on the protective of CTS. And then the type of macrophage polarization was measured. As Figure 5C shown, compound C (5 μM) treatment effectively blocked the phosphorylation of AMPK. Moreover, the expression of iNOS and CD86 were inhibited and the expression of Arg-1 and CD206 were augmented by CTS, which were deprived following compound C stimulation (Figure 5D). Flow cytometry results also revealed that compound C inhibited CTS induced M2 type macrophage and promoted polarization of macrophages to M1 type (Figure 5E). These results suggest that AMPK was closely involved in the regulation of CTS on macrophage polarization.

### 3.6. CTS regulates RAW264.7 macrophage metabolism by activating AMPK-HIF-1α

**FIGURE 6:**
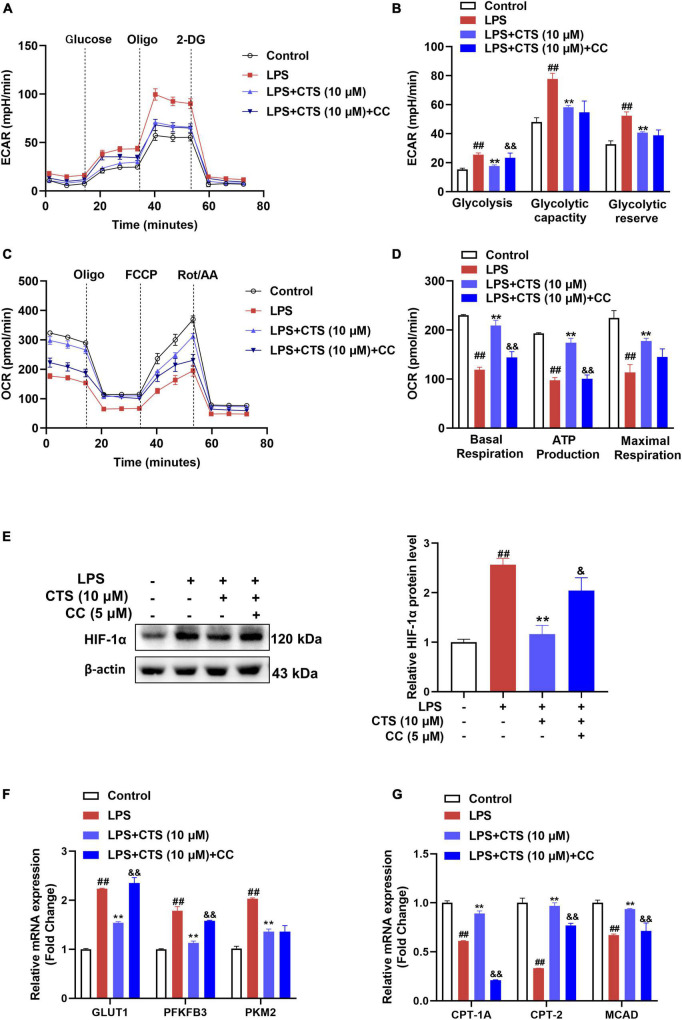
Cryptotanshinone (CTS) regulates energy metabolism of LPS-induced RAW264.7 macrophages through AMPK. RAW264.7 pretreated with 5 μM AMPK inhibitor compound C for 2 h were treated with 10 μM CTS for 2 h and then exposed to LPS for another 24 h. **(A)** ECAR of the indicated macrophages were measured with a seahorse analyzer. **(B)** Glycolysis, glycolytic capacity, and glycolytic reserve were calculated and are indicated as ECAR in mpH/min, *n* = 4. **(C)** OCR was measured with the Seahorse analyzer. **(D)** The basal respiration, maximal respiration, and ATP production were calculated and are indicated as OCR in pmoles/min, *n* = 4. **(E)** The protein level of HIF-1α was analyzed by using western blotting assay, *n* = 3. **(F)** The mRNA expressions of glucose transporter type1 (GLUT1), 6-phosphofructo-2-kinase/fructose-2,6-biphosphatase 3 (PFKFB3), pyruvate kinase M2 (PKM2) were detected by using the qPCR assay, *n* = 3. **(G)** The mRNA expressions of carnitine palmitoyltransferase 1A (CPT1A), carnitine palmitoyltransferase 2 (CPT2), medium-chain acyl-coA dehydrogenase (MCAD) were detected by using the qPCR assay, *n* = 3. Data were presented as the mean ± SEM. ^##^*P* < 0.01 vs. the control group; *P < 0.05, **P < 0.01 vs. the LPS group; ^&^*P* < 0.05, ^&&^*P* < 0.01 vs. the LPS + CTS (10 μM) group.

Since changes of macrophage polarization phenotype are closely related to cell metabolism, we further explored whether AMPK is involved in the regulation on metabolism of macrophage. Our results showed that LPS sharply increased rate of glycolysis and deterioration of mitochondrial oxidative phosphorylation of macrophage, while CTS antagonized these results (Figures 6A–D). However, inhibition of AMPK partially blocked the effects of CTS on macrophage metabolism dysfunction. There is ample evidence that AMPK acts as a negative regulator of the “Warburg” effect, by inhibiting HIF-1α-mediated glycolysis (39–41). The LPS-induced metabolic switch from OXPHOS to aerobic glycolysis in macrophages is associated with HIF1α activation (42). This metabolic pattern switch depends on the stabilization of HIF-1α and resulting in in the expression of key glycolytic proteins, such as GLUT1, PKM2, and PFKFB3 (43). Here, our results showed that LPS significantly promoted the expression of HIF-1α, which was ameliorated by CTS treatment (Figure 6E). However, inhibition of AMPK by compound C blocked the effect of CTS on HIF-1α and the mRNA expression of GLUT1 and PFKFB3 (Figures 6E, F). FAO, an important provider of acetyl CoA that fuels the TCA cycle and OXPHOS, is significantly increased in M2 macrophages (44). AMPK is a key regulator of FAO, which promotes the increase of intracellular FAO by increasing the expression of fatty acid metabolism enzymes, such as carnitine palmitoyltransferase 1A (CPT1A), carnitine palmitoyltransferase 2 (CPT2) and medium-chain acyl-coA dehydrogenase (MCAD) (45, 46). Therefore, we speculated that CTS enhanced mitochondrial oxidative phosphorylation through activation of AMPK-mediated increases in FAO. The effect of CTS on the expression of fatty acid oxidation genes were determined. Our results showed that the expressions of CPT1A, CPT2 and MCAD were significantly inhibited by LPS while promoted by CTS (Figure 6G). However, Compound C blocked the mRNA expression of CPT1A, CPT-2 and MCAD. These results suggested that AMPK was involved in role of CTS on regulation of macrophage polarization and metabolism reprogramming.

## 4. Discussion

ALI is a diffuse inflammatory injury of lung parenchyma caused by a variety of non-cardiac internal and external lung pathogenic factors, which is clinically manifested as respiratory failure, hypoxemia and pulmonary edema with high morbidity and mortality (47, 48). In addition, ALI has been considered to be an important factor causing the death of critically ill patients with The Corona Virus Disease 2019 (COVID-19) that is currently circulating worldwide (49, 50). A growing body of evidence indicates that the key pathogenesis of ALI is cytokine storm induced by immune cell. Too much proinflammatory cytokines caused inflammation of the lungs and serious destruction of the alveolar capillary barrier, further resulting in the decrease of lung compliance and a sharp deterioration in pulmonary function (51, 52). Therefore, inhibiting excessive inflammatory response in the lung is a key strategy for the treatment of ALI. However, to date, the treatment strategies for acute lung injury are limited and no drugs targeting inflammatory responses during ALI have been approved. ALI patients can only rely on supportive strategies to save their lives (53). Therefore, it is urgent to develop new and effective drugs to treat patients with acute lung injury.

As the main component of the cell wall of gram-negative bacteria, LPS could activate the *in vivo* innate immune system and induce pulmonary inflammatory responses, and was used to simulate ALI syndrome *in vivo* (54). In this study, intratracheal infusion of LPS was used to induce acute lung injury model of rat and the pulmonary function was rapidly destroyed as indicated by obvious diffuse alveolar injury, excessive infiltration of inflammatory cells and pulmonary edema. CTS is one of the main biologically active ingredient of *salvia miltiorrhiza* with powerful anti-inflammatory activity and higher distribution in the lung tissue (15, 55). Given its anti-inflammatory property and tissue-specific distribution in lung, CTS may be a potential compound for the treatment of acute lung injury. According to our previous results, the dosages of CTS used in this study were 15, 30 and 60 mg/kg/day (19). In this study, our results firmly showed that CTS restored pulmonary function of rats in a dose-dependent manner as indicated by the improved lung compliance and alveolar capillary barrier integrity.

Alveolar macrophages are the most abundant immune cells in the lung tissue and are crucial for maintaining airway homeostasis (56). Different phenotypes of macrophage played different roles during the pathological process of acute lung injury (56). Activation of pro-inflammatory (M1) phenotype macrophage is a key parameter of acute pneumonia, which stimulates cytokine storm by releasing proinflammatory factors such as IL-1β, IL-6 and TNF-α (57). In contrast, activation of the anti-inflammatory (M2) phenotype macrophages ameliorates lung tissue injury by releasing anti-inflammatory mediators Arg-1 and IL-10 to promote inflammation resolution (58). Therefore, modulation of macrophage polarization is a potentially effective treatment for acute lung injury (58).

CTS has been shown to be able to convert M1 to M2 phenotype macrophages to alleviate ulcerative colitis lesions and significantly inhibit neuroinflammation in ischemic stroke (20, 21). This suggests that CTS may be an immunomodulator targeting macrophage polarization to treat inflammatory diseases. Consistently, our results showed that CTS was able to inhibit the activation of M1 phenotype macrophage and promote the activation of M2 phenotype macrophage during acute lung injury. Additionally, metabolic reprogramming of macrophage plays a key role in the process of macrophage polarization (31). M1 macrophage showed reduced oxidative metabolism and increased glycolysis, while M2 macrophage has a complete tricarboxylic acid cycle and utilizes FAO and OXPHOS for energy supplement (35). The glycolytic inhibitor 2-DG inhibits the activation of M1 type macrophage, whereas viral knockdown of the fatty acid-related gene CPT1A inhibited activation of M2 type macrophage (12, 13, 59). This suggests that blocking or restoring metabolic pathways to modulate macrophage polarization is feasible. Consistent with previous study, we found that CTS inhibited LPS-induced glycolysis and promoted mitochondrial oxidative phosphorylation of macrophage (22, 23). These results firstly revealed that CTS exerted its protective effects by regulating macrophage polarization and metabolism reprogramming.

Previous studies suggested that AMPK acted as a key protein molecule in regulating metabolism reprogramming and polarization of macrophage by repressing the expression of HIF-1α (39, 41). Activation of AMPK could promote transformation of macrophages from the pro-inflammatory type to the anti-inflammatory type (36). Consistent with previous reports, the activation of AMPK was inhibited with LPS stimulation both *in vivo* and *in vitro* (37), which was relieved by CTS in a dose-dependently manner. Previous studies have shown that HIF-1α-mediated glycolysis drove macrophage differentiated into the pro-inflammatory phenotype (60). Furthermore, the M2 phenotype polarization of macrophage was dependent on AMPK-induced increase in FAO (10), including the up-regulation of FAO related enzymes such as CPT1A, CPT2 and MCAD (45, 61). In this study, compound C, an AMPK specific inhibitor, blocked CTS-induced inhibition of HIF-1α and the expression of key glycolysis genes (such as GLUT1 and PFKFB3), subsequently abrogating CTS-induced promotion of key FAO enzyme genes (e.g., CPT1A, CPT2, and MCAD). These findings suggested that the ability of CTS to induce macrophages polarization was mainly attributed to the activation of AMPK to induce FAO and inhibit HIF-1α-mediated glycolysis.

However, the mechanism by which CTS activates AMPK remains unknown. Liver kinase B1 (LKB1) is one of the upstream kinases that regulate AMPK activation and directly phosphorylates Thr172 of the α subunit of AMPK to activate AMPK. Studies have shown that LPS reduced AMPK phosphorylation in macrophages and inhibited LKB1 activation (62). It has been reported that CTS activates AMPK signaling pathway by LKB1 (63). Therefore, it was reasonable to speculate that CTS might promote AMPK phosphorylation through activation of LKB1, but this needs further experimental verification.

In conclusion, our study suggested that CTS promoted the transformation of M1-type macrophages into M2-type macrophages by regulating energy metabolism, thus playing an anti-acute lung injury role. Furthermore, we have shown that AMPK mediated the regulation of CTS on macrophage polarization by affecting energy metabolism patterns of macrophage.

## Data availability statement

The original contributions presented in this study are included in the article/Supplementary material, further inquiries can be directed to the corresponding authors.

## Ethics statement

This animal study was reviewed and approved by Sun Yat-sen University Animal Ethics.

## Author contributions

ZY, PW, and GF: research design, performed experiments, data collection, and writing—original draft. QW and CL: assisted the research and data collection. JL, PL, and JC: project administration, funding acquisition, and writing—review and editing. All authors have read the author agreement of the magazine and agreed to the published version of the manuscript.
