# Supplementary material for: Cryptotanshinone attenuates LPS-induced acute lung injury by regulating metabolic reprogramming of macrophage
Source: Front Med (Lausanne). 2023 Jan 13;9:1075465. doi: 10.3389/fmed.2022.1075465 (PMC9880059; doi:10.3389/fmed.2022.1075465)
Supplement: Supplementary file 1 [file Presentation_1.PPTX]

## Slide 1
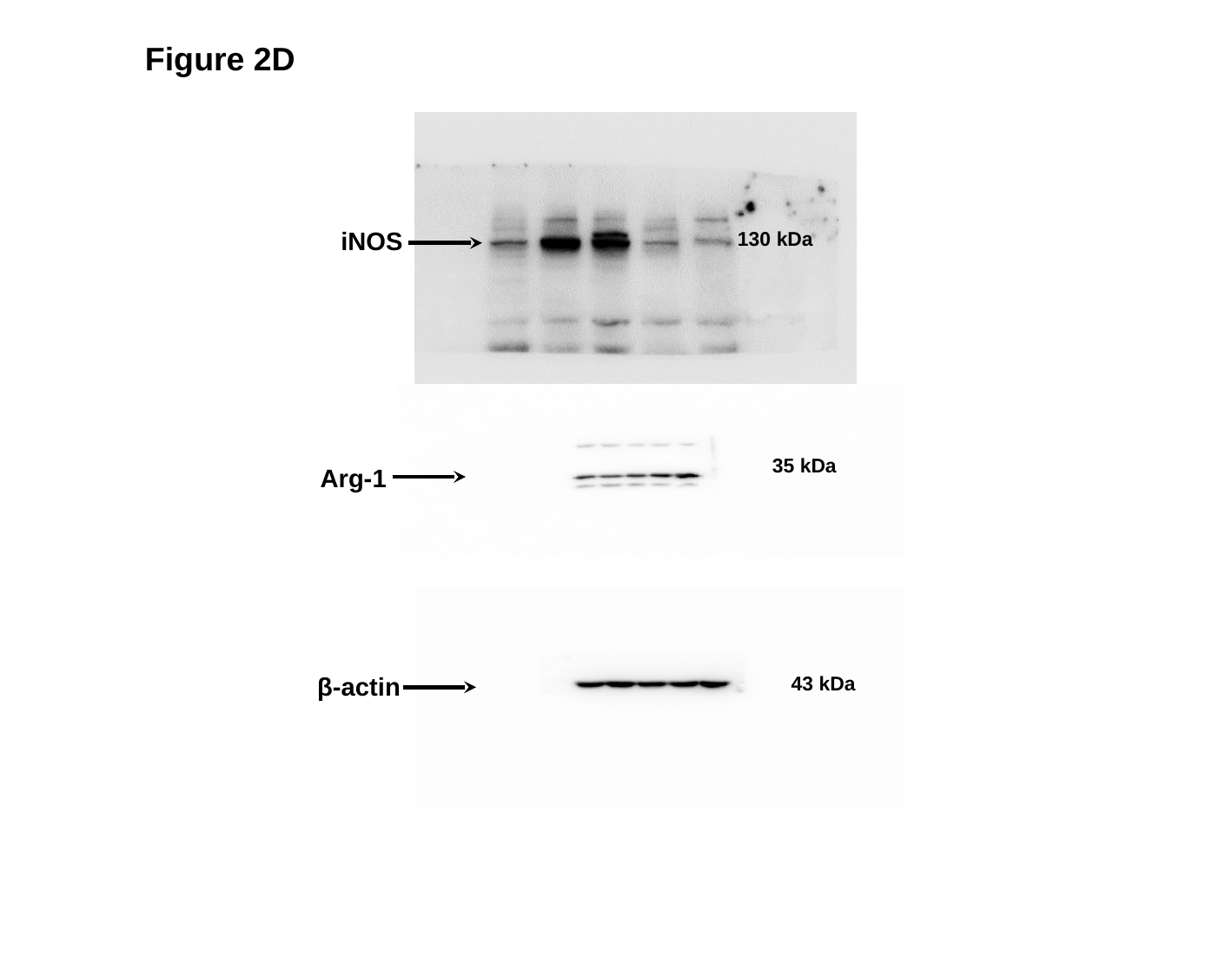

Figure 2D
iNOS
130 kDa
35 kDa
Arg-1
43 kDa
β-actin

## Slide 2
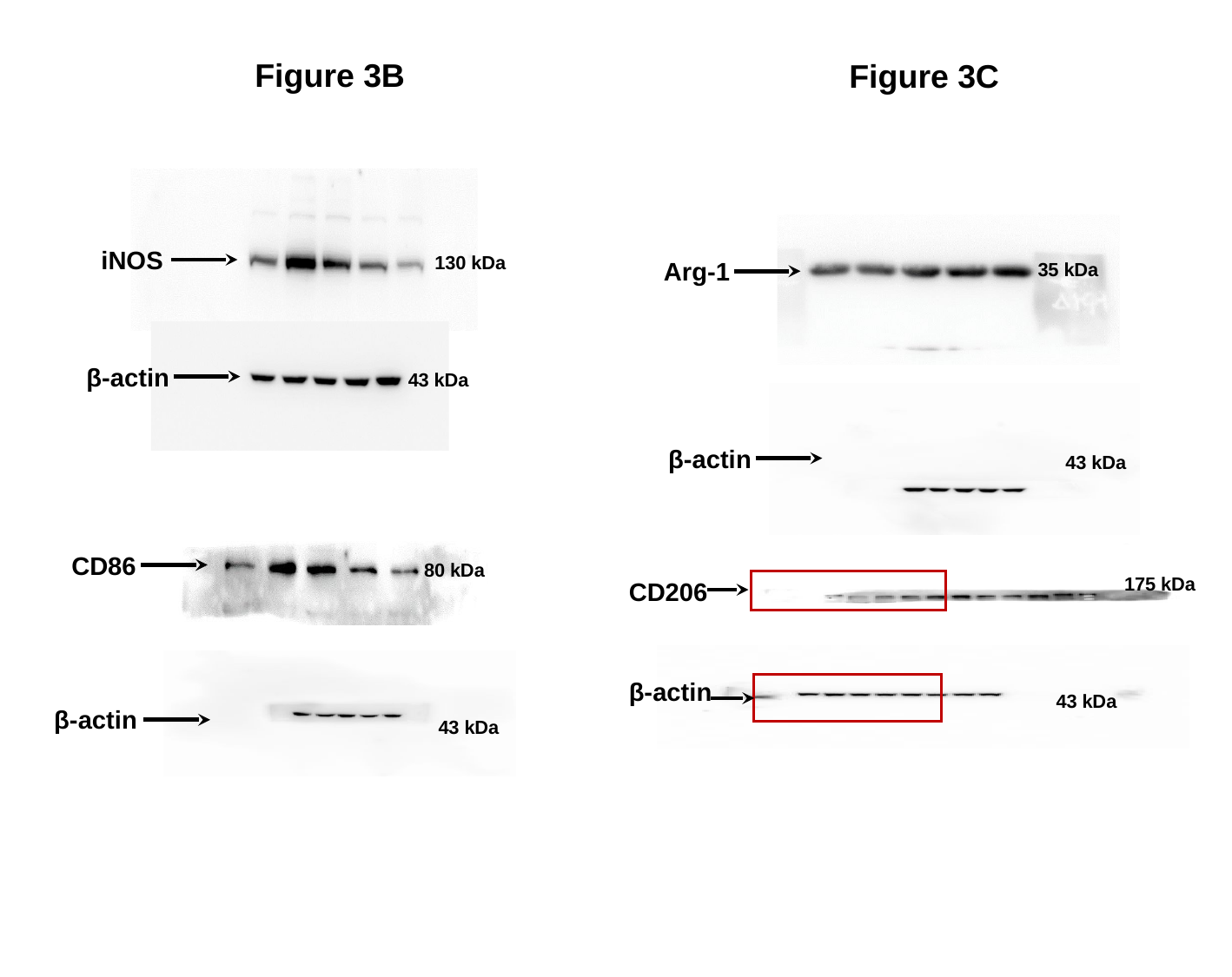

Figure 3B
Figure 3C
iNOS
130 kDa
Arg-1
35 kDa
β-actin
43 kDa
β-actin
43 kDa
CD86
80 kDa
175 kDa
CD206
β-actin
43 kDa
β-actin
43 kDa

## Slide 3
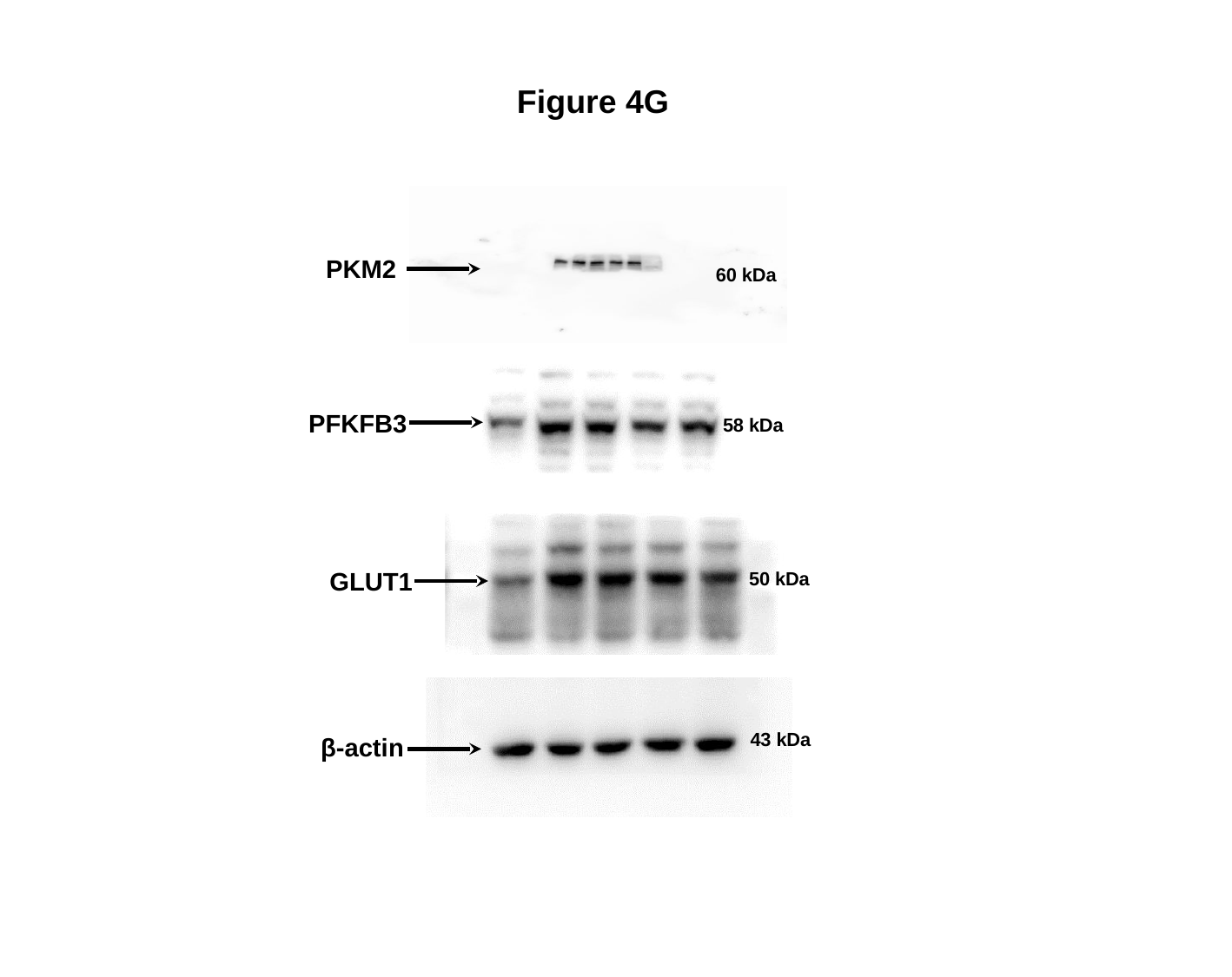

Figure 4G
PKM2
60 kDa
PFKFB3
58 kDa
GLUT1
50 kDa
43 kDa
β-actin

## Slide 4
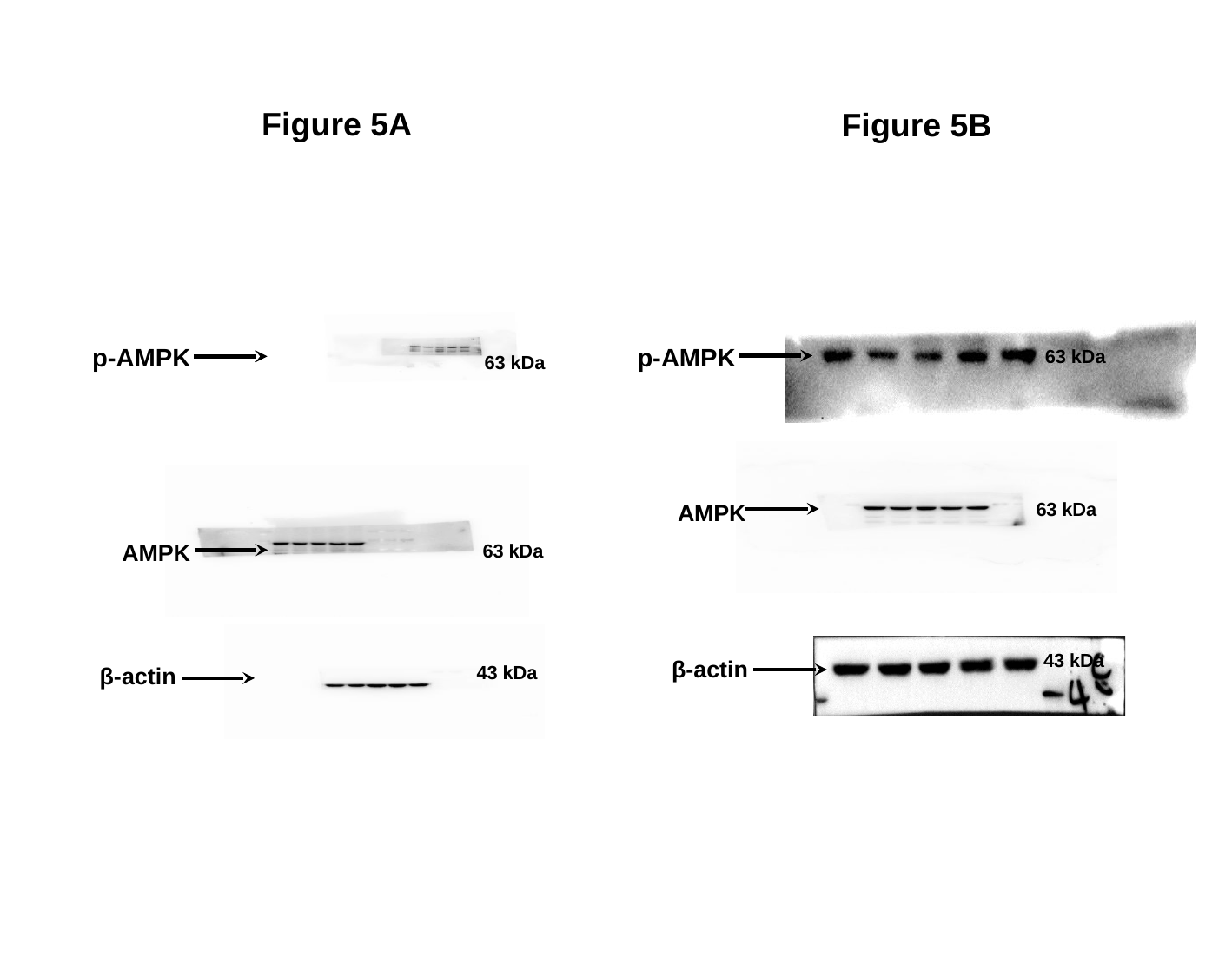

Figure 5A
Figure 5B
p-AMPK
p-AMPK
63 kDa
63 kDa
63 kDa
AMPK
AMPK
63 kDa
43 kDa
β-actin
43 kDa
β-actin

## Slide 5
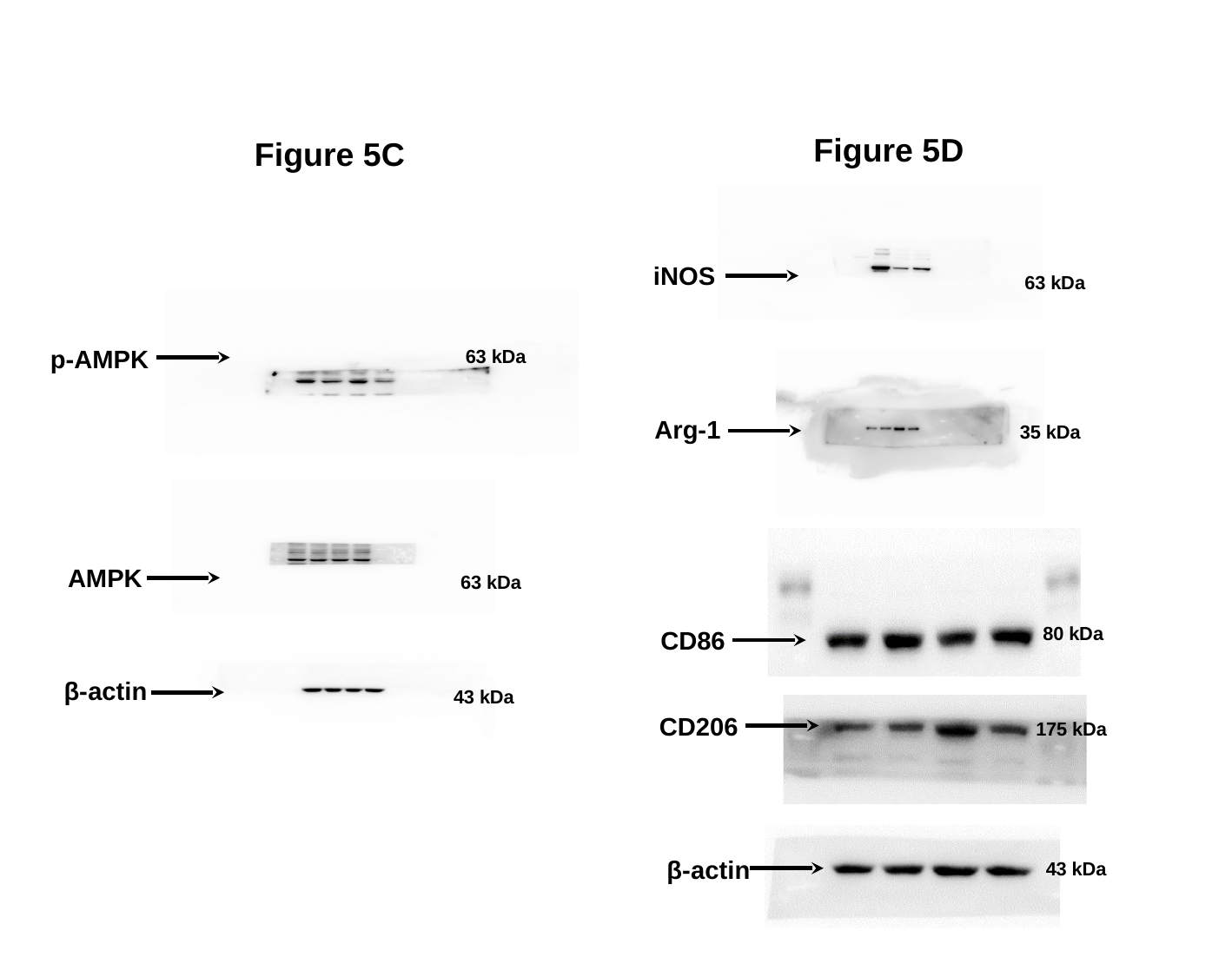

Figure 5D
Figure 5C
iNOS
63 kDa
p-AMPK
63 kDa
Arg-1
35 kDa
AMPK
63 kDa
80 kDa
CD86
β-actin
43 kDa
CD206
175 kDa
β-actin
43 kDa

## Slide 6
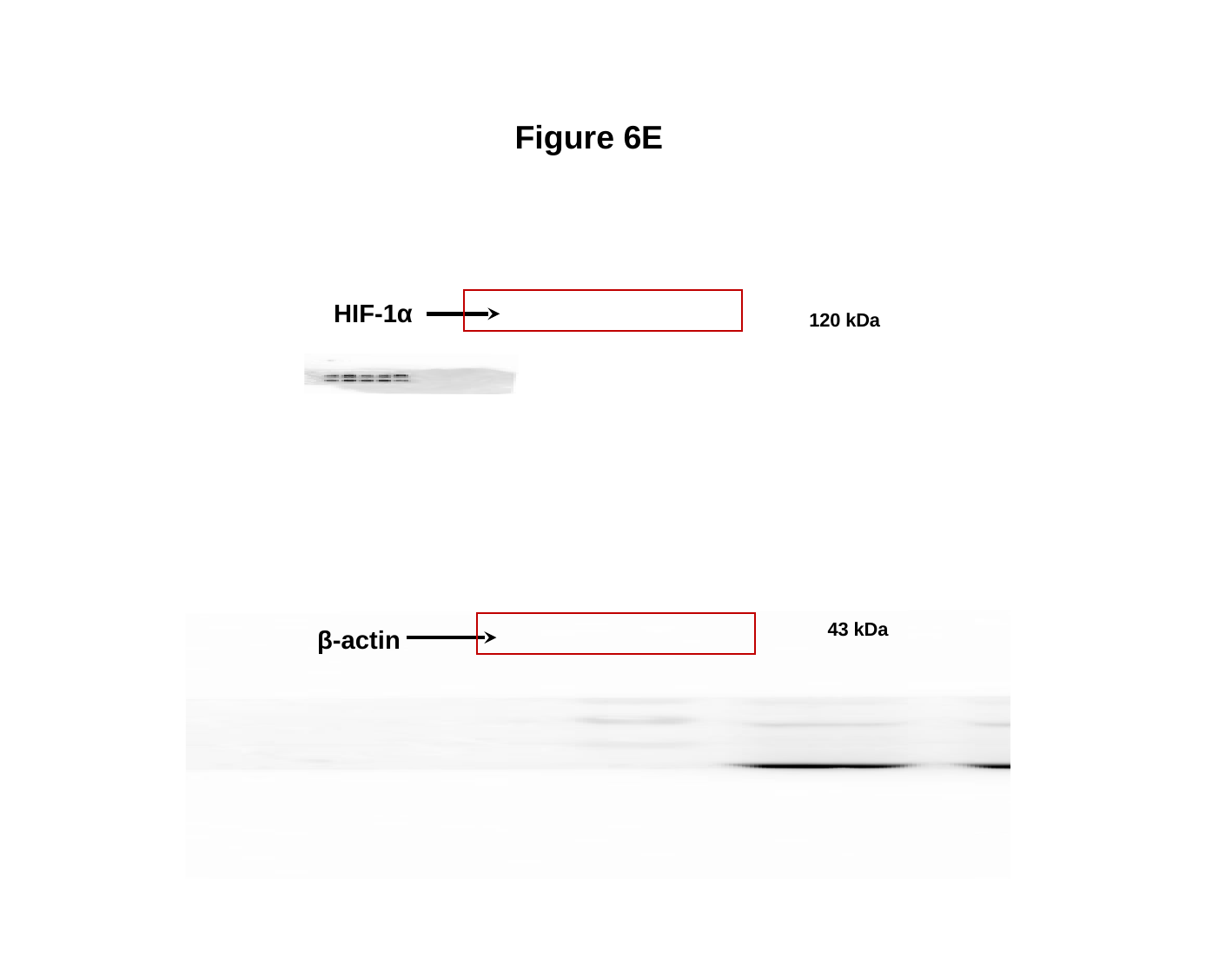

Figure 6E
HIF-1α
120 kDa
β-actin
43 kDa
